# Resident Education and Virtual Medicine: A Faculty Development Session to Enhance Trainee Skills in the Realm of Telemedicine

**DOI:** 10.15766/mep_2374-8265.11302

**Published:** 2023-03-07

**Authors:** Anna E. Rueda, Ana C. Monterrey, Margaret Wood, Linessa Zuniga, Betty Del Rio Rodriguez

**Affiliations:** 1 Assistant Professor, Division of Academic General Pediatrics, Department of Pediatrics, Baylor College of Medicine

**Keywords:** Telehealth, Telemedicine, Virtual Learning, Pediatrics, Case-Based Learning, Faculty Development

## Abstract

**Introduction:**

COVID-19 accelerated the use of telemedicine. Subsequently, clinical sites began conducting virtual visits. Academic institutions implemented telemedicine for patient care and simultaneously had to teach residents the logistics and best practices. To meet this need, we developed a training session for faculty focused on telemedicine best practices and teaching telemedicine in the pediatric realm.

**Methods:**

We designed this training session based on institutional and society guidelines and faculty experience with telemedicine. Objectives included telemedicine documentation, triage, counseling, and ethical issues in telemedicine. We conducted all sessions in a 60-minute or 90-minute format over a virtual platform for small and large groups using case scenarios with photos, videos, and interactive questions. A novel mnemonic ABLES (awake-background-lighting-exposure-sound) was created to guide providers during the virtual exam. Following the session, participants completed a survey evaluating content and presenter effectiveness.

**Results:**

We presented the training sessions between May 2020 and August 2021 to 120 participants. Participants included pediatric fellows and faculty, reaching 75 participants locally and 45 nationally (at Pediatric Academic Society and Association of Pediatric Program Directors meetings). Sixty evaluations (response rate: 50%) showed favorable results for general satisfaction and content.

**Discussion:**

This telemedicine training session was well received by pediatric providers and addressed the need for training faculty to teach telemedicine. Future directions include adapting the training session for medical students and developing a longitudinal curriculum that applies telehealth skills learned with patients in real time.

## Educational Objectives

By the end of this activity, learners will be able to:

1. Define telehealth and telemedicine.
2. Discuss the teaching challenges and opportunities that arise when utilizing telehealth in a pediatric learning environment.
3. Model how to triage what pediatric patient visits are appropriate for telemedicine.
4. Articulate the basic requirements for documenting and billing a telemedicine encounter.
5. Apply the ABLES (awake-background-lighting-exposure-sound) mnemonic to teaching a pediatric virtual physical exam.
6. Explain best practices for maintaining confidentiality during a virtual visit with an adolescent.

## Introduction

The COVID-19 pandemic accelerated the use of telemedicine as a health care delivery platform in March 2020, and many clinical sites began conducting virtual visits routinely.^1^ When the pandemic started, academic institutions needed not only to quickly implement the use of telemedicine for the care of patients but simultaneously to teach residents the logistics and best practices as well. Telemedicine offers many advantages to pediatric patient care, such as increased access to care for families in rural areas and those with limited transportation or childcare for siblings; reduced travel for complex, technology-dependent patients; and increased ease of follow-up for patients requiring frequent visits. Given these advantages, it is critical that pediatricians in academic or other teaching environments have access to training to gain the skills needed to teach the next generation of pediatricians the best practices in performing telemedicine visits. At the onset of the COVID-19 pandemic, most health care providers had not received any formalized training in telemedicine^2^ or how best to teach these new skills in the clinical environment. Numerous *MedEdPORTAL* educational materials have been developed targeting telemedicine education to preclinical medical students^3,4^ and students during clerkships.^5^ Fewer curricula are specifically aimed at pediatric graduate medical education and teaching learners in the clinical environment.^6,7^

Therefore, to meet this need, we developed a training session for faculty that focuses on telemedicine best practices and teaching telemedicine in the pediatric realm. The skills gained in this training session are intended specifically for use in the outpatient pediatric setting and can be applied to both general pediatrics and subspecialty visits.

## Methods

### Theoretical Foundation

We created a brief training session on how to teach telemedicine in the outpatient pediatric setting that can be applied to both primary care and subspecialty visits. We based this training session on adult learning principles as described by Knowles^8^ to make the material relevant and timely. Knowing that adults are self-directed and have a great wealth of experience they can utilize in solidification of their knowledge, we made the material as interactive as possible with a case-based format.

### Needs Assessment and Instructional Design

As mentioned above, the COVID-19 pandemic created a heavy reliance on telemedicine that had previously not existed. As frontline providers at an academic training site, we quickly realized that there was a need for instruction not only in how to perform telemedicine visits but also in how to educate learners in carrying out these visits. To identify relevant content to include, we reviewed various sources published in the literature, including medical society and education guidelines from the American Academy of Pediatrics,^9^ the American College of Physicians,^10^ and the Association of American Medical Colleges.^11^ We also included material covering some self-identified gaps in knowledge, such as being familiar with institutional and state telehealth and documentation requirements, coaching parents through a virtual exam for pediatric patients, and ethical considerations like adolescent privacy.^12,13^ We reviewed our own institutional guidelines in conducting telemedicine encounters and leveraged our personal experience conducting telemedicine visits in primary care teaching clinics with resident trainees since the very start of the pandemic.

### Instructional Methods

The session incorporated interactive elements within the small- and large-group settings to engage the audience and facilitate discussion and application of best practices. We created five cases highlighting important aspects of conducting a telehealth visit, including triaging patients, conducting a virtual physical exam, counseling, and ethical considerations like confidentiality. We also created the ABLES mnemonic teaching card based on our own personal experience conducting telehealth physical exams with patients. ABLES stands for awake-background-lighting-exposure-sound. This tool was a reference to guide learners in the important elements of a successful virtual physical exam and was incorporated into the presentation when discussing the virtual exam. It could be shared electronically or printed for attendees (see Appendix A: ABLES Teaching Card).

### Audience and Facilitator Requirements

We first implemented the training session at our institution to teach subspecialty fellows and faculty who worked with and taught resident trainees (see Appendix B: Teaching Material With Presenter Notes). This helped address the urgent need for providers at various levels to be competent in conducting telemedicine visits with residents. We presented the first session at our institution's pediatric educational retreat in a 60-minute format, followed by several invited talks for the department. We also presented the material in a 90-minute workshop format at two national conferences after peer-reviewed submission. Based on an iterative practice, we reviewed and updated the materials after each presentation to further optimize the education delivered and relevance to the audience. Prerequisite knowledge was not required of attendees, but as part of the session, we elicited attendees’ experience with telehealth to tailor the discussion to their level of educational need. The session would be best conducted by one to two facilitators who have reviewed the content in detail to prepare. Our facilitators were all academic primary care pediatricians using telehealth in the clinical setting. Future facilitators should have experience with telehealth visits and knowledge about how these visits are conducted at their own institution to ensure the ability to answer questions and guide discussion.

### Delivery of Instruction

We delivered the training session via a virtual platform due to the ongoing COVID-19 pandemic, but it could also be delivered in person in a location with the ability to project PowerPoint slides, show videos with audio, and arrange attendees in small groups. We delivered the training session in a 60-minute or 90-minute format (see Appendix C: Sample Timeline). For 90-minute sessions, we used virtual breakout rooms to discuss each case in small groups and then reported back key learning points to the larger group. For 60-minute sessions, we limited discussion time for each case and used the live chat function to discuss learning points as a large group. Electronic handouts (or hard copies if in person) could be provided to attendees in advance to facilitate discussion questions and to reference the resources reviewed during the session (see Appendix A: ABLES Teaching Card).

Session implementation included (1) delivering content verbally while sharing and advancing slides, (2) monitoring the chat discussion if done virtually, and (3) facilitating discussions in breakout rooms or small groups (see Appendix D: Facilitator Guide). Depending on the size of the audience, multiple facilitators could be helpful to join each breakout room or rotate in and out of breakout rooms to ensure that discussions are productive. Similarly, if conducted in person, at least one facilitator should circulate between discussion groups.

### Session Evaluation

To ensure we achieved our teaching goals, we developed our evaluation strategy using the objectives for each session. We used a 5-point Likert scale (1 = *poor,* 5 = *excellent*) to evaluate how well each objective was met, the utility of content for attendees, and the effectiveness of the presenters. Free-text responses measured attendees’ self-reported changes in practice and solicited feedback on changes to future sessions. Learners completed the evaluation postsurvey electronically via REDCap. The facilitators shared the link for the survey via the virtual chat box, a quick response (QR) code on a PowerPoint slide, or email after completion of the activity. If conducted in person, hard copies of the evaluation could be provided to attendees at the end of the session if preferred. See Appendix E: Session Evaluation for a copy of the evaluation postsurvey.

## Results

The training session reached 120 participants of whom 75 were from the local institution and included faculty and fellows with teaching responsibilities. Nationally, the training session reached 45 participants: 30 participants at the 2021 Association of Pediatric Program Directors annual meeting and 15 at the 2021 Pediatric Academic Societies annual meeting. We presented the training session in workshop format for the national conferences to facilitate discussion and exchange of ideas. The audience was mainly pediatric faculty from other academic institutions. In total, we received 60 evaluations for a collective response rate of 50%—a 68% response rate when presented locally and a response rate of approximately 23% from national presentations. Evaluation results were favorable for general satisfaction and content (average: 4.5–4.7; Table 1). The teaching-specific objectives scores were high (average: 4.6–4.8; Table 1), highlighting the strengths of the training session in presenting useful information on the practice of telemedicine. We elicited comments from the participants to evaluate how the training session would change their practice of telemedicine (Table 2). One of the most common themes elicited was the incorporation of learners into the practice of telemedicine. Participants mentioned how the training session helped them with ideas and prepared them to integrate teaching in real time while doing telemedicine. Also, participants found it useful to address ethical concerns, including confidentiality when treating adolescents. The ABLES mnemonic was found by participants to be valuable to help guide and prepare learners before and during the virtual encounter. We adapted and improved the training session with each presentation, incorporating recommendations from previous participants.

**Table 1.**
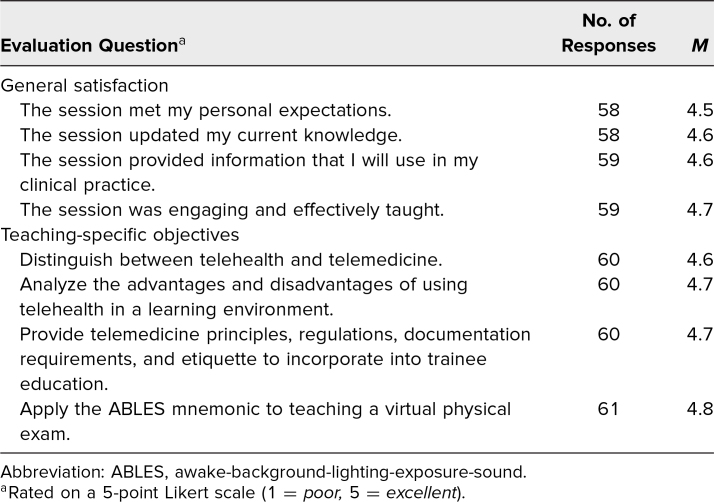
Evaluation Results From Telemedicine Training for Pediatric Educators

**Table 2.**
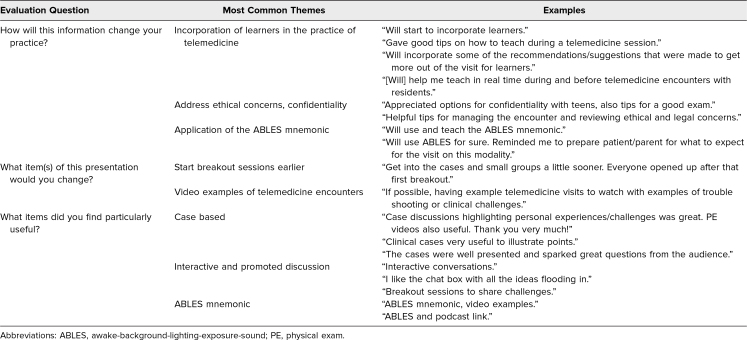
Telemedicine for Pediatrics: Most Common Themes on Comments Elicited From Training Session Evaluation

## Discussion

We initially developed this content as a case-based lecture for pediatric residents. Shortly afterwards, we were approached by faculty at our institution to develop it into a faculty development training session since, at the time of development, there was a paucity of education on telemedicine pediatric best practices and how to incorporate trainees, especially residents. Our training session expanded upon medical society and educational guidelines and included real-life experiences of academic general pediatricians using telehealth with resident trainees. Our approach was well received by participants, with the majority agreeing the session was timely and useful.

As mentioned, this session was presented several times to different audiences both locally and nationally. After presenting locally, we revised the documentation and billing aspects of the content to make it more generalizable to the national audience and less specific to our own state's billing requirements and our institution's electronic medical record system. Participant feedback on the initial iterations of the training helped us to adapt the order of the cases and content, the better to engage future participants in interactive learning earlier in the training. We also ordered the cases to mimic the flow of a virtual case, for example, starting with a triage case and ending with a case focused on how to maintain confidentiality.

We received many positive comments on the use of interactive cases to present the materials. Participants found the virtual physical exam tips and guidance on communication during the visit valuable. The ABLES teaching card is a novel tool created by our group for this training session to aid in remembering how to best prepare learners for an effective virtual physical exam. We anticipate that with the continued use of telemedicine, the ABLES tool can be utilized in undergraduate and graduate medical education and by providers as a pocket card to aid in conducting a proper and thorough virtual exam. Participants specified on several occasions that they appreciated discussion and tips surrounding sensitive history taking and options for confidentiality with adolescents. Participants also benefited from discussing how to prepare for a telemedicine visit with learners and how to best guide them through triaging a video visit, the logistics of the visit, and documentation. One aspect of the training session that should not be overlooked and that was mentioned several times by participants is the value in having multiple perspectives from and participation by the other members of the small and/or large groups. Utilizing the knowledge and experience shared during these discussions allowed participants to learn from each other and develop solutions in real time for implementing telehealth teaching practices in their own setting.

Through this experience, we found a great need for additional education in this area for both providers and trainees. While the training session was generally found to be useful, the evaluation data collected were based on self-report only. Therefore, we did not directly assess whether the learners achieved the stated objectives or implemented what they had learned into their future practice of teaching telemedicine. In addition, some participant feedback asked for further help with troubleshooting the use of specific telehealth platforms and requested more detailed information on billing requirements. The varied platforms used for telemedicine made this a difficult aspect to include in a general training session. We provided introductory guidance on documentation and billing. However, we kept this information general as each institution and state might approach them differently. As a result, the specifics on these topics could not be taught to a national audience. We also did not include training on how to incorporate interpreters into telemedicine encounters; this is also a topic that could be included in future training.

We envision providers continuing to use this resource to teach trainees about telemedicine, as the session touches on points not traditionally taught in clinical practice, such as general telehealth documentation, virtual exam techniques, and incorporation of technology into day-to-day clinical practice. Additional aspects of telemedicine that could be explored include access to care and ethics of telemedicine as well as specific institutional instruction on billing, coding, and troubleshooting.

Finally, this training session was developed during the early stages of the COVID-19 pandemic for practitioners with minimal to no experience in conducting telemedicine encounters with pediatric patients. Practitioners with vast experience could find the training session somewhat introductory. However, we feel this training meets the needs of those educators who may not yet have an established telehealth training session and helps to outline pediatric-specific guidelines for teaching telehealth to pediatric residents.

## Appendices


ABLES Teaching Card.pdfTeaching Material With Presenter Notes.pptxSample Timeline.docxFacilitator Guide.docxSession Evaluation.docx
*All appendices are peer reviewed as integral parts of the Original Publication.*
