# Supplementary material for: Resident Education and Virtual Medicine: A Faculty Development Session to Enhance Trainee Skills in the Realm of Telemedicine
Source: MedEdPORTAL. 2023 Mar 7;19:11302. doi: 10.15766/mep_2374-8265.11302 (PMC9989055; doi:10.15766/mep_2374-8265.11302)
Supplement: Supplementary file 1 — ABLES Teaching Card.pdfTeaching Material With Presenter Notes.pptxSample Timeline.docxFacilitator Guide.docxSession Evaluation.docx [file mep_2374-8265.11302-s001.zip › MEP-2022-0090/A. ABLES Teaching Card.pdf]

HOW TO BE "ABLES" TO PERFORM A PEDIATRIC TELEHEALTH EXAM

Awake

- Mental status and behavior are strong determinants of severity of illness
- Patient must be present and awake to complete the visit

Background

- History and chief complaint can assist you in targeting areas of interest
- Includes history of present illness and history from chart review

Lighting

- In the room of the patient's home
- Extra light, flashlight, or phone light to examine darker areas, i.e. inside the mouth

Exposure

- Part to be examined
- Adjust the camera for your needs
- Patient/parent can complete specific tasks

Sound

- Must be functional to coordinate with visual exam
- Eliminate background sounds as much as possible

A. Rueda, B. Del Rio Rodriguez, A. Monterrey, M. Wood and L. Zuniga. 2022.

Texas Children's Hospital
Baylor College of Medicine
