# Supplementary material for: Resident Education and Virtual Medicine: A Faculty Development Session to Enhance Trainee Skills in the Realm of Telemedicine
Source: MedEdPORTAL. 2023 Mar 7;19:11302. doi: 10.15766/mep_2374-8265.11302 (PMC9989055; doi:10.15766/mep_2374-8265.11302)
Supplement: Supplementary file 1 — ABLES Teaching Card.pdfTeaching Material With Presenter Notes.pptxSample Timeline.docxFacilitator Guide.docxSession Evaluation.docx [file mep_2374-8265.11302-s001.zip › MEP-2022-0090/C. Sample Timeline.docx]

**Appendix C. Sample Timeline for Session: Resident Education and Virtual Medicine**

| **90-min total** | **60-min total** |  |
| --- | --- | --- |
| 5 min | 5 min | **Introduction and Objectives:**  Participants also introduce themselves and their roles |
| 15 min | 5 min | **Case #0:**  Gauge learner levels/experiences, telehealth and telemedicine didactic |
| 15 min | 10 min | **Case #1:**  Preparing learning and triaging appropriate visits for telemedicine using *Rash* case example  *Option for breakout to small groups to discuss triage for video vs in-person visits* |
| 10 min | 5-8 min | **Case #2:**  History taking and documenting in telemedicine using *ADHD* case example |
| 15 min | 10 min | **Case #3:** Physical exam techniques in telemedicine and introduction of the ABLES mnemonic using *Cough and fever* case example  *Option for breakout to small groups to troubleshoot physical exam techniques used over video* |
| 10 min | 5-8 min | **Case #4:**  Virtual counseling using *Lifestyle Counseling* case example |
| 15 min | 10 min | **Case #5:**  Ethical dilemmas using *Adolescent with dysuria* case example  *Option for breakout to small groups to discuss confidentiality strategies* |
| 5 min | 5 min | **Wrap-up:**  Summary of main points and time for questions  Complete post-survey evaluation |
