# Supplementary material for: Resident Education and Virtual Medicine: A Faculty Development Session to Enhance Trainee Skills in the Realm of Telemedicine
Source: MedEdPORTAL. 2023 Mar 7;19:11302. doi: 10.15766/mep_2374-8265.11302 (PMC9989055; doi:10.15766/mep_2374-8265.11302)
Supplement: Supplementary file 1 — ABLES Teaching Card.pdfTeaching Material With Presenter Notes.pptxSample Timeline.docxFacilitator Guide.docxSession Evaluation.docx [file mep_2374-8265.11302-s001.zip › MEP-2022-0090/D. Facilitator Guide.docx]

Appendix D: Facilitator Guide

Resident Education and Virtual Medicine:

A Faculty Development Session to Enhance Trainee Skills in the Realm of Telemedicine

**Slides 0-4** (5 min)

Introduction

- Presenters introduce themselves and may ask participants (depending on the size of the audience) to introduce themselves and the role they serve in trainee education.
- Review the Goal and Objectives as written.

**Slides 5-6** (5 min)

Introductory Case, Preparing the learner

- Use this case as an icebreaker to obtain baseline knowledge from audience, including current use of telemedicine, current clinic workflow, and troubleshooting techniques. You may ask someone in the audience to read the case if time permits.
- Lead the group through the discussion questions, using the notes provided with this slide.

**Slides 7-14** (10 min)

Telehealth/Telemedicine definitions, current practice in telemedicine, and using telemedicine in medical education

- These slides go over basic concepts and definitions in Telehealth and Telemedicine.
- Slide 8 Feel free to adapt the information on CMS (Centers for Medicare and Medicaid Services) if you know it has changed at the time of the presentation.
- Slide 9 This figure showcases the different areas of patient care delivery within Telemedicine and provides some examples.
- Before you go to slide 10, ask the audience in what areas of telemedicine learners can be incorporated.
- Slide 10 Shows the areas to incorporate the learner. Feel free to provide examples of how learners are incorporated during synchronous and asynchronous encounters.
- Slide 11 This is an opportunity for the participants to share how they are using telemedicine.
- Slide 12 Ask the questions to the audience.
- Slide 13 and 14 summarize challenges and advantages.

**Slides 15-18** (5 min)

Preparing learners to perform a virtual exam

- This section is intended to help in the preparation of the learner to conduct a telemedicine encounter, considering the type of encounter, the virtual platform used and other processes that need to take place before the actual visit. Use the slide notes to direct your teaching conversations for each slide

**Slides 19-23** (15 min)

Case #1 Triaging Rash

- Read the prompt and ask the group to consider the listed questions during the small group break out. ​
- You may consider providing the questions to the group separately via email or other document since slides will not be available during virtual breakouts. ​
- For in person learning, you can provide hard copies of the questions for the group to reference.
- **Small Group Break Out: Use 5-10 min for the group to discuss**
- **Large Group Discussion**: Have a member from each small group to share what they discussed. Use the slide notes to ensure you emphasize the main learning points, if the group does not bring them up on their own.
- On the summary slide of this case, you may consider summarizing these points once more to ensure the group has understood the main teaching concepts to glean from this case.

**Slides 24-34 (15 min)**

Case #2 Documentation and Billing

- The case is to be read to the participants to set the stage for discussion on proper documentation for Telemedicine encounters.
- This case focuses on a follow up for ADHD and emphasizes important teaching points for supervising faculty on necessary documentation for a video visit.
- Use the slide notes to take the participants through this case.
- Although billing for visits may differ by state or institution, the last slide includes good general points about billing, and has a list of helpful resources for participants, which the facilitator can refer to in the presentation.

**Slides 35-43 (15 min)**

Case #3 The Virtual Physical Exam

- Read the case to the participants to start or ask for a volunteer to read.
- Depending on the modality of your presentation (virtual or in-person), you will want to encourage participation for the next few slides. You can mention to have the video on, use the chat box, raise their hand or call out their answers.
- Emphasize to the participants to not to focus on identifying a diagnosis, but on pros/cons of the videos. Encourage them to think of how they would teach a resident about how to best obtain a virtual exam for this patient.
- The first video is the “what not to do”, poor example. Use the slide notes to facilitate your learners to identify the main pros and cons of this video example.
- The second video is the better example. Encourage your participants to call out what they notice is improved, or alternatively type in the chat box for a virtual lecture.
- Review the important portions of a physical exam that can be performed virtually and use the slide notes to guide your discussion (Slides 39-40).
- **Small Group Discussion**: Allow 5 minutes. Break into small groups. Ask the participants discuss the questions on the slide 41.
- **Large Group Discussion**: A member from each small group will briefly share what their group thought about the questions.
- Introduce participants to the “ABLES” mnemonic and how it can be used in practice.
- You can have this printed out or emailed to the group for future reference.

**Slides 44-47 (10 min)**

Case #4 Virtual Counseling

- This case focuses on the learning concept that virtual visits offer a great opportunity for preceptors to directly observe learners counseling skills.
- Start the case by asking participants how they would guide the learner in approaching this type of case prior to starting a video visit.
- Guide the participants through the main points of the case and the practice questions using the slide notes.
- On the final slide of this case (Slide 47) reiterate the importance of understanding and using motivational interviewing techniques and resources that can be used to improve MI skills.

**Slides 48-53 (10 min)**

Case #5 Confidentiality and Standard of Care

- Read the question aloud to start this case.
- Discuss importance of maintaining standard of care regarding confidentiality when treating teens for certain medical issues. Using slide notes, guide them through these concepts.
- **Small Group Discussion**: Break into small groups for approximately 5 minutes. Prior to break out, ask the participants to consider the questions on slide 49.
- **Large Group Discussion:** Participants rejoin the large group and a member from each small group is invited to share what was discussed in their group during the breakout.
- Review the specific example of how to establish rapport and introduce the importance of a teen patient’s confidentiality in telemedicine. List options of how to keep an interview confidential on a video visit. Finally, using slide notes from Slide 51-53, teach about using professional judgement when approaching ethical issues and deciding if an in-person visit is more appropriate.

**Slides 54-57 (2 min)**

- Summary, resources, and evaluation slides
- You may update or add references
- Add a QR or link to the evaluation or provide paper evaluation.
