# Supplementary material for: Resident Education and Virtual Medicine: A Faculty Development Session to Enhance Trainee Skills in the Realm of Telemedicine
Source: MedEdPORTAL. 2023 Mar 7;19:11302. doi: 10.15766/mep_2374-8265.11302 (PMC9989055; doi:10.15766/mep_2374-8265.11302)
Supplement: Supplementary file 1 — ABLES Teaching Card.pdfTeaching Material With Presenter Notes.pptxSample Timeline.docxFacilitator Guide.docxSession Evaluation.docx [file mep_2374-8265.11302-s001.zip › MEP-2022-0090/E. Session Evaluation.docx]

Appendix E. Resident Education and Virtual Medicine:

Presentation Evaluation

Please help us improve this session by completing the evaluation below.

**PROGRAM OBJECTIVES**

Please indicate how well this session achieved each of the following objectives by choosing ONE option for EACH item:

*At the conclusion of the activity, the participants should be able to:*

|  | Poor | Fair | Average | Good | Excellent |
| --- | --- | --- | --- | --- | --- |
| Define telehealth and telemedicine. |  |  |  |  |  |
| Discuss the teaching challenges and opportunities that arise when utilizing telehealth in a pediatric learning environment. |  |  |  |  |  |
| Model how to triage what pediatric patient visits are appropriate for telemedicine. |  |  |  |  |  |
| Articulate the basic requirements for documentation and billing a telemedicine encounter. |  |  |  |  |  |
| Apply the ABLES mnemonic to teaching a pediatric virtual physical exam. |  |  |  |  |  |
| Explain best practices for maintaining confidentiality during a virtual visit with an adolescent. |  |  |  |  |  |

**ABOUT THIS ACTIVITY**

Please indicate the overall effectiveness of this activity by indicating your level of agreement with the following. Please check ONE option for EACH item.

*This activity:*

|  | Strongly Disagree | Disagree | Neutral | Agree | Strongly Agree |
| --- | --- | --- | --- | --- | --- |
| Met my personal expectations |  |  |  |  |  |
| Updated my current knowledge |  |  |  |  |  |
| Provided information that I will use in my clinical practice. |  |  |  |  |  |
| Was engaging and effectively taught. |  |  |  |  |  |

How will this information change your practice?

What item(s) of this presentation would you change?

What items did you find particularly useful?
